# Production of *Dermatophagoides farinae* Having Low Bacterial Content Using Ampicillin

**DOI:** 10.1155/2023/9024595

**Published:** 2023-05-18

**Authors:** Ju Yeong Kim, Myung-hee Yi, Myungjun Kim, Jun Ho Choi, Seogwon Lee, Tai-Soon Yong

**Affiliations:** ^1^Department of Environmental Medical Biology, Institute of Tropical Medicine and Arthropods of Medical Importance Resource Bank, Yonsei University College of Medicine, Seoul 03722, Republic of Korea; ^2^Institute of Allergy, Yonsei University College of Medicine, Seoul, Republic of Korea

## Abstract

**Background:**

Symbiotic bacteria in house dust mites pose a risk of immunological side effects in the clinical use of immunotherapeutic agents. In this study, we investigated the duration for which the bacterial concentration in *Dermatophagoides farinae* could be kept low with antibiotic treatment, and whether the allergenic properties of the mite changed under ampicillin treatment.

**Methods:**

*D. farinae* was cultivated in the presence of ampicillin powder in an autoclaved medium for 6 weeks. After subsequent subcultures without ampicillin, the mites were harvested, and the extract was prepared. The amounts of bacteria, lipopolysaccharides (LPS), and two major allergens (Der f 1 and Der f 2) were measured. Human bronchial epithelial cells and mice were treated with the *D. farinae* extract to assess the allergic airway inflammation.

**Results:**

The number of bacteria and level of LPS were reduced by 150-fold and 33-fold, respectively, at least 18 weeks after ampicillin treatment. The concentration of Der f 1 and Der f 2 remained unchanged by ampicillin treatment. The secretion of interleukin (IL)-6 and IL-8 from the human airway epithelial cells decreased when treated with the extract of ampicillin-treated *D. farinae* compared with that of ampicillin-untreated *D. farinae*. A mouse asthma model was developed using ampicillin-treated *D. farinae*. We observed that the level of lung function, airway inflammation, and serum-specific immunoglobulin were not different for the mouse asthma model developed using ampicillin-treated *D. farinae* than the model developed using ampicillin-untreated *D. farinae*.

**Conclusions:**

We showed that bacterial content in *D. farinae* was reduced by ampicillin treatment, which was sufficient to induce allergic sensitization and an immune response. This method will be used to develop more controlled allergy immunotherapeutic agents.

## 1. Introduction

The house dust mite (HDM) is a major perennial allergen source that plays a pivotal role in the pathogenesis of allergic diseases [1, 2]. Sensitization to mite allergens in the first years of life has a significant clinical effect on lung function and is associated with long-term clinical outcomes of respiratory health [3]. In fact, about 50% of asthmatics are allergic to HDM [4]. More than 30 HDM allergens have been identified as fundamental triggers in allergic reactions, and bacterial substances in HDM correlated with asthma risk and severity [5, 6].

A series of studies have revealed the presence of Gram-negative bacteria in HDM [7–9]. Whole-genome shotgun sequencing of *Dermatophagoides farinae* revealed that *Enterobacter* was the most abundant bacterium [10], while another study using 16S ribosomal (r)-RNA cloning reported that *Bartonella* was the most abundant bacterial taxon [7]. In our previous study, using high-throughput sequencing technology, we validated that *D. farinae* contains *Bartonella* as the core bacterium and small *Enterococcus* [8, 9]. Further, another HDM species, *D. pteronyssinus*, was confirmed to contain *Klebsiella pneumoniae* in our previous study, although the amount was very low [8]. A recent study showed that *Cardinium* is associated with *D. farinae* strains from the USA, China, and Europe, which may play biological roles in *D. farinae* [11].

Bacterial substances in HDM can act as a trigger for the development of allergic immune responses in the innate immune function of airway epithelial cells via pattern recognition receptors such as toll-like receptors (TLR) and nucleotide-binding oligomerization domain-containing proteins (NODs) [12, 13].

Lipopolysaccharide (LPS) is derived from bacteria present in HDM and has been well characterized as immunomodulatory substances [12]. In addition, experimental studies have suggested that exposure to LPS levels may be associated with the induction of allergic responses [14, 15]. Further, LPS has also been measured in *D. farinae* and *D. pteronyssinus*. While *D. farinae* showed a relatively high LPS concentration, *D. pteronyssinus* contained almost no LPS [8, 16]. Overall, the difference in LPS levels between *D. farinae* and *D. pteronyssinus* can be due to the occurrence of *Cardinium* or *Bartonella* in *D. farinae*.

Using antibiotics to develop more controlled allergy immunotherapeutic agents is believed to be a useful strategy to reduce the risk of bacterial substances abundantly present in *D. farinae*. In this study, we aimed to determine whether treatment with ampicillin, a broad-spectrum antibiotic, during *D. farinae* cultivation, would reduce the presence of symbiotic bacteria and substances derived from them, such as LPS, in *D. farinae*. The change in the bacterial amount in *D. farinae* was investigated during multiple passages of *D. farinae* cultivation after one antibiotic treatment to ensure that one antibiotic treatment was sufficient to maintain a low level of bacterial amount in *D. farinae* for multiple *D. farinae* generations. Furthermore, we measured the concentrations of major allergens (i.e., Der f 1 and Der f 2) after antibiotic treatment, because allergen expression may be influenced by changes in the microbiome composition of the storage mite [17]. We also evaluated the usefulness of our bacteria-reduced HDM extract by examining the *in vivo* allergenicity of antibiotic-treated HDM extract in an asthma mouse model.

## 2. Materials and Methods

### 2.1. Ethics Statement

All animal studies were approved by the Laboratory Animal Resources Committee (number: 2018-0316) of Yonsei University College of Medicine.

### 2.2. Mite Cultivation and Protein Extraction

Inactive dry yeast (Choheung Co., Gyeonggi, South Korea) was mixed with fish food (KKW1Q0040; Jeil Feed Co., Daejeon, South Korea) in a ratio of 1 : 1, and the mixture was autoclaved. *D. farinae* were previously collected from beds in a Korean household in 1998. Mites were cultivated in autoclaved medium in T75 cell culture flasks (70075; SPL Life Sciences, Gyeonggi, South Korea) at 25°C. We decided to use 1% ampicillin to reduce bacteria in HDM because a pilot study revealed that 1% ampicillin did not affect HDM growth. The ampicillin-treated *D. farinae* group was cultivated in the presence of 1% ampicillin powder (0.05 g of ampicillin powder and 5 g of food mixture; Georgia Chemical & Equipment Co., Inc., Norcross, GA, USA) in autoclaved medium at 25°C for 6 weeks, a standardized cultivation period [18–20]. Subsequent subcultures were performed in a fresh autoclave medium without ampicillin every 6 weeks. After 18 weeks, the mites were harvested with saturated saline and used to prepare a homogenate by sonication at 500 W, 10 times for 10 s with 10 s pauses, and centrifuged at 10,000 *× g* for 30 min. Foreign substances, including bacteria, were removed from the extracts using a sterile 0.22 *µ*m pore-sized Millex-GP syringe filter (EMD Millipore, Burlington, MA, USA). The extracts used for both *in vitro* and *in vivo* experiments were produced using the mites cultured for 18 weeks after ampicillin treatment.

### 2.3. Real-Time Polymerase Chain Reaction

Total genomic DNA was isolated from house dust mites obtained at each subculture using the commercially available NucleoSpin DNA Insect Kit (Macherey-Nagel, Duren, Germany). Primer set Bact1369/Prok1492 was used for the quantification of total bacteria by real-time PCR [21] on an Applied Biosystems Step One™ Real-Time PCR System (Applied Biosystems, USA) using SybrGreen as the fluorescent dye. The sequences of the primers were as follows: 16S rRNA forward primer (BACT1369F): 5′-CGGTGAATACGTTCYCGG-3′, 16S rRNA reverse primer (PROK1492R): 5′-GGWTACCTTGTTACGACTT-3′, *D. farinae* elongation factor 1-*α* forward primer (EF1*α*F): 5′-ACCCGTGAACATGCTTTGCT-3′, *D. farinae* elongation factor 1-*α* reverse primer (EF1*α*R): 5′-CACCATTCTCTCAAGCTCGT-3′. Relative total bacteria DNA was quantified using the *ΔΔ*Ct method and normalized to elongation factor of *D. farinae*. For each sample, triplicate reactions were analyzed.

### 2.4. Lipopolysaccharide Measurement

The concentration of LPS in the ampicillin-treated *D. farinae* and untreated *D. farinae* extracts were measured using the LAL QCL-1000 Kit (Lonza, Basel, Switzerland).

### 2.5. Concentration of the Der f 1 and Der f 2 Measurement

The concentration of Der f 1 and Der f 2 in mites was measured using the enzyme-linked immunosorbent assay (ELISA) kit (INDOOR Biotechnologies, Charlottesville, VA, USA) according to the manufacturer's instructions.

### 2.6. Cell Culture and Cytokine Measurement by Enzyme-Linked Immunosorbent Assay

Human bronchial epithelial cells (BEAS-2B, ATCC® CRL-9609™, Rockville, MD, USA) were obtained from the American Type Culture Collection. BEAS-2B cells were maintained in Dulbecco's Modified Eagle Medium/F40 medium supplemented with 10% inactivated fetal bovine serum at 37°C and 5% carbon dioxide. HDM was used to treat cells seeded at a concentration of 1 × 10^6^ cells/well in six well plates [20]. Cells were sampled 24 hr after a single exposure to 100 *μ*g/mL each of the ampicillin treated-*D. farinae* and untreated *D. farinae* extracts. The amount of cytokine produced in the medium was measured using Quantikine human interleukin (IL)-6 and IL-8 ELISA kits (R&D Systems, Minneapolis, MN, USA) according to the manufacturer's instructions. Three independent samples were examined following exposure to mite extracts or phosphate-buffered saline (PBS) as a control.

### 2.7. Mouse Asthma Model

Mouse models were constructed using a previously described model of allergic asthma [22]. Mice were housed under specific pathogen-free conditions and a 12 hr light–dark cycle. Female BALB/c mice (8 weeks old) were purchased from Orient Bio (Seongnam, Korea). There were three groups of mice: mice sensitized and challenged with ampicillin-treated *D. farinae* (D.f. + AMP, *n* = 5), mice sensitized and challenged with the untreated *D. farinae* extract (D.f., *n* = 5), and the control group (*n* = 5). On days 0 and 14, the mice were sensitized with mite extract by intraperitoneal injection. One hundred micrograms of mite extract (D.f. or D.f. + AMP) were resuspended in 2 mg of alum. On day 24, the mice were challenged for three consecutive days (days 24, 25, and 26). The mite extracts of D.f. and D.f. + AMP groups (30 *μ*g) were resuspended in PBS, and 50 *μ*L of the solution was administered to the mice intranasally. Control mice received 50 *µ*L of sterile PBS. Airway inflammation assessments were performed 24 hr after the last challenge.

### 2.8. Airway Inflammation Assessments

Airway hyperresponsiveness (AHR) was measured 24 hr after the last treatment using the FlexiVent system (Scireq Inc., Montreal, QC, Canada), as previously described [23]. Bronchoalveolar lavage (BAL) fluid was collected, and differential cell counting was performed as previously described [23]. Histological analysis of the lung tissue using hematoxylin and eosin and periodic acid-Schiff staining was performed as previously described [23].

### 2.9. Measurement of the *Dermatophagoides farinae*-Specific Immunoglobulin

Wells of microtiter ELISA plates were individually coated with 100 *μ*L of *D. farinae* extracts (2 *μ*g/mL). *D. farinae*-specific immunoglobulin (Ig)-G1, IgG2a, and IgE levels in mouse serum were measured using ELISA [23]. Briefly, 50 *µ*L of diluted sera were added to each well and incubated overnight at 4°C. Then, the wells were washed with a wash buffer (0.05% Tween-20 in PBS) and incubated with the appropriate antibody for 2 hr. Secondary antibodies were detected using biotinylated anti-mouse IgE (1 : 1,000; 408804; BioLegend, San Diego, CA, USA), biotinylated goat anti-mouse IgG1 (1 : 10,000; NBP1-69914B; Novus Biologicals, Littleton, CO, USA), and biotinylated goat anti-mouse IgG2a (1 : 10,000; NBP1-69915B; Novus Biologicals). The signal was developed using 3,3′, 5,5′-tetramethylbenzidine after streptavidin–peroxidase treatment. The optical density was measured at a wavelength of 450 nm using VersaMax (Molecular Devices, Seoul, South Korea). Data were normalized to those of the PBS exposure control group.

### 2.10. Immunohistochemistry

Tissue sections were heated for 20 min in FLEX Target Retrieval Solution, Low pH 6.0 (Dako K8005). Sections were incubated in 3% H_2_O_2_ for 5 min at room temperature followed by two high-volume washes with TBS for 5 min per wash. Sections were then incubated in primary goat antibody (1 : 10,000 anti-intercellular adhesion molecule-1 (ICAM-1), 1 : 1,000 anti-vascular cell adhesion protein-1 (VCAM-1); R&D Systems, Minneapolis, MN, USA) for 1 hr. After a high-volume wash with TBS, sections were incubated in anti-goat biotinylated secondary antibody (1 *µ*g/mL, Vector Laboratories, Burlingame, CA) for 45 min. Sections were then incubated for 20 min with horseradish peroxidase-conjugated avidin (Elite kit, Vector Laboratories) and were washed thrice in a large volume of TBS for 15 min per wash. A diaminobenzidine (DAB) kit was then used to detect the antigens by incubating the sections with DAB for 5 min. After being rinsed in distilled deionized water, sections were counterstained with hematoxylin, rinsed in distilled water, and dehydrated in graded alcohols and xylene before being coverslipped and mounted with Permount.

### 2.11. Statistical Analysis

All results are expressed as the mean ± standard error of the mean. Multivariate data of AHR were evaluated for group differences using two-way analysis of variance (ANOVA) followed by Bonferroni's post hoc test. The remaining data were analyzed using Student's *t*-test or ANOVA, followed by Bonferroni's post hoc test. Statistical analysis was performed using the GraphPad Prism software (version 9.0; GraphPad, Inc., La Jolla, CA, USA). Statistical significance was set at *p* < 0.05.

## 3. Results

### 3.1. Reduction of the Bacterial Content of *Dermatophagoides farinae* by Ampicillin Treatment

**Figure 1:**
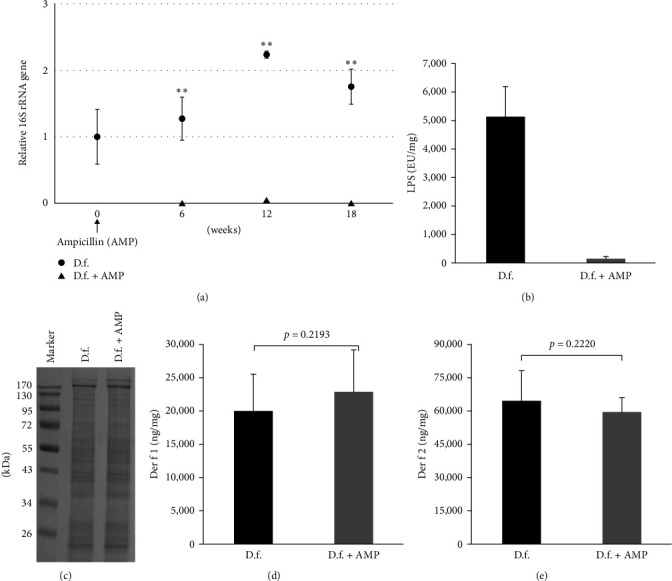
Reduction of the bacterial content of *Dermatophagoides farinae* by ampicillin (AMP) treatment. (a) Relative amounts of bacterial 16S rRNA gene in ampicillin-treated *D. farinae* (D.f. + AMP) and untreated *D. farinae* (D.f.). (b) Lipopolysaccharide (LPS) concentration in the ampicillin-treated and untreated *D. farinae* extracts. (c) Protein pattern of the extracts from the ampicillin-treated and untreated *D. farinae* groups. Concentration of Der f 1 (d) and Der f 2 (e) in *D. farinae* extracts.  ^*∗∗*^*p* < 0.01.

RT-PCR confirmed that the antibiotic treatment reduced the bacterial 16S rRNA gene level in *D. farinae*, which did not increase during the three passages of cultivation (6 weeks for one passage, Figure 1(a)). The bacterial amount was 150-fold lower in the ampicillin-treated group than in the untreated group after three passages of cultivation following ampicillin treatment (Figure 1(a)).

Thereafter, we assessed the change in the immunological characterization of HDM extracts after three passages of cultivation following ampicillin treatment. The concentrations of LPS in ampicillin-treated and untreated *D. farinae* were 155.6 and 5,135.5 EU/mg, respectively (Figure 1(b)). However, the protein patterns in gel electrophoresis and allergen (Der f 1 and Der f 2) concentration in the mite extract did not differ between the ampicillin-treated *D. farinae* and untreated *D. farinae* groups (Figures 1(c) and 1(d).

### 3.2. Allergic Airway Inflammation Induction Using Ampicillin-Treated-*D. farinae*

**Figure 2:**
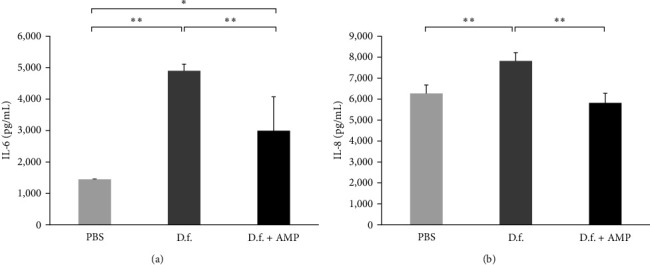
Inflammatory cytokine (a) interleukin (IL)-6 and (b) IL-8 secretions in airway epithelial cells (BEAS-2B) of the ampicillin treated-*Dermatophagoides farinae* (D.f. + AMP) and untreated *D. farinae* (D.f.) groups. Data are presented as the mean ± standard deviation of at least three independent experiments. PBS, phosphate-buffered saline.  ^*∗∗*^*p* < 0.01,  ^*∗*^*p* < 0.05.

**Figure 3:**
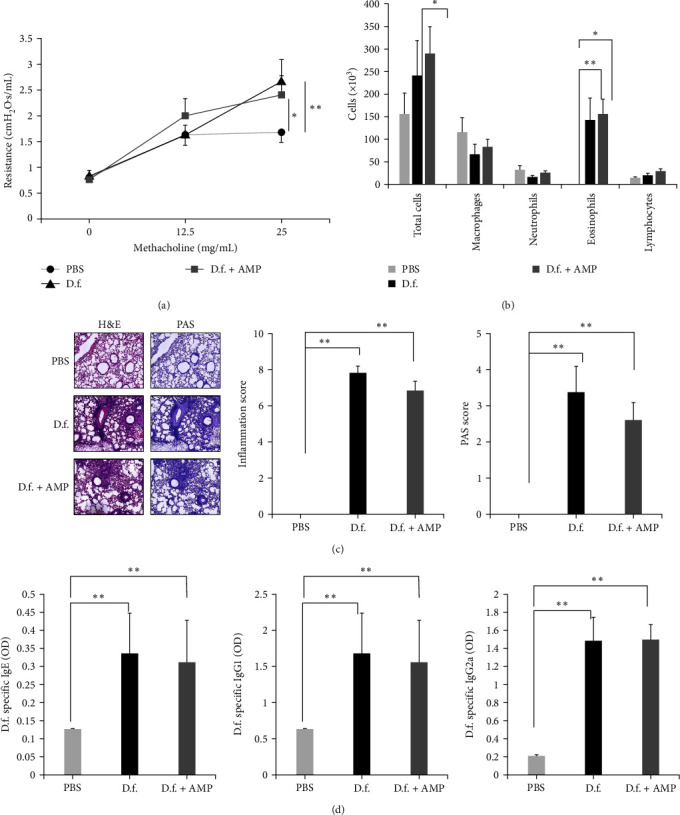
Induction of airway hyperreactivity with ampicillin treated-*Dermatophagoides farinae* (D.f. + AMP) and untreated *D. farinae* (D.f.) *in vivo*. (a) Airway resistance values. (b) Inflammatory cell infiltration in the lung. (c) Hematoxylin and eosin (H&E), periodic acid-schiff (PAS), and Masson's trichrome staining of the lung sections. (d) Expression of *D. farinae*-specific immunoglobulin (Ig)-E, IgG1, and IgG2a in mice. Data are presented as the mean ± standard deviation of at least three independent experiments. PBS, phosphate-buffered saline.  ^*∗∗*^*p* < 0.01,  ^*∗*^*p* < 0.05.

Levels of IL-8 and IL-6 were significantly reduced in cells treated with ampicillin-treated *D. farinae* compared with those with untreated *D. farinae* (Figures 2(a) and 2(b). Next, we developed an airway allergic inflammation model to compare the allergenicity of ampicillin-treated and untreated *D. farinae in vivo*. Allergic inflammation was induced in both the ampicillin-treated and untreated *D. farinae* groups. Both the groups showed increased airway resistance (Figure 3(a)), a BAL eosinophil count (Figure 3(b)), inflammatory cell infiltration, mucus production in the lung (Figure 3(c)), and serum *D. farinae*-specific IgE, IgG1, and IgG2a levels (Figure 3(d)). In addition, immunohistochemistry revealed increased ICAM-1 and VCAM-1 expression in vessels in the lung tissues in mice with both ampicillin-treated *D. farinae* and untreated *D. farinae*-induced asthma (Figure S1). There was no significant difference in these measured parameters between the ampicillin-treated and untreated *D. farinae* groups.

## 4. Discussion

In this study, we found that a single ampicillin treatment was sufficient to maintain low levels of bacteria in HDM for at least 18 weeks. In addition, LPS concentration in the extract was markedly reduced in ampicillin-treated *D. farinae*. In this study, the LPS concentrations in ampicillin-treated and untreated *D. farinae* were 155.6 and 5,135.5 EU/mg, respectively. A previous study reported that LPS concentrations were 8,740 EU/mg in Korean *D. farinae* and 3,890 EU/mg in a commercially available *D. farinae* extract in the United States [24]. In addition, the HDM extract had the same protein pattern in gel electrophoresis and concentration of the major allergen (Der f 1 and Der f 2) as that in the HDM extract cultured without ampicillin (Figure 1). We also showed that the secretion of proinflammatory cytokines, IL-6 and IL-8, decreased when human airway epithelial cells were treated with antibiotic-treated HDM (Figure 2), providing evidence that the bacteria in HDM had an effect on the immune response.

Previous studies have reported that different dust mite species have different compositions and amount of the microbiome in their bodies [7–10]. One previous study using PCR reported that *D. farinae* contained *Bartonella* and other Gram-negative species, and the authors suggested that these bacteria are likely to be the sources of the LPS found in the mite allergenic extract [7]. Furthermore, in our previous microbiome study of *D. farinae* and *Tyrophagus putrescentiae*, both mites contained Gram-negative bacteria, such as *Bartonella*, and bacteria-derived LPS [8]. We also showed that *D. farinae* possessed greater bacterial concentration and LPS than *D. pteronyssinus* and that the immune response pathway against bacteria was activated only in *D. farinae*-treated human airway cells [20].

*Cardinium* was detected in *D. farinae* strains in China, Europe, and the USA [11]. In addition, a recent study reported that *Cardinium* might modulate mite gene expression related to immunity and metabolism [25]. Similarly, *Wolbachia*, the intracellular symbiotic bacteria in *T. putrescentiae*, may play biological roles [11, 26–29]. Thus, changes in these bacteria may affect the production of allergens.

LPS can induce the development of type 2 immune responses to the inhaled allergens via TLR4 signaling [30, 31]. In addition, LPS is reportedly responsible for determining the development and severity of allergic diseases, such as asthma [15, 32]. The LPS of HDM acts as a TLR4 trigger of the airway epithelial cells and induces allergic inflammation through the activation of mucosal DCs [12]. However, some types of LPS produced by certain bacteria species such as *Bartonella quintana* have an antagonistic effect on TLR4 [33]. Therefore, it is necessary to consider which microbiomes exist in the mite to estimate the immunotherapeutic efficacy of the extract.

Although it is unlikely that the commercially available HDM extract for AIT contains live bacteria, bacterial antigens are present therein. A study reported that HDMs may serve as carriers of the bacteria responsible for the induction of IgE sensitization to microbial antigens [34]. In addition, bacterial IgE sensitization has been associated with severe atopic dermatitis and comorbidity of rhinitis. Moreover, IgE sensitization to bacteria has shown to have clinical relevance in allergic diseases [35–40]. Therefore, we believe that if the effect on AIT is maintained, it is better to reduce the bacteria in HDMs.

Furthermore, a recent mouse asthma model revealed that HDM-associated Gram-negative bacteria aggravated disease severity through nucleotide-binding oligomerization domain-containing protein 1 (NOD1) [13]. Their results showed that recognition of peptidoglycan caused both NOD1 and NOD2 to self-oligomerize, resulting in nuclear factor-kB activation and transcription of multiple inflammatory genes. In addition, they found the presence of some muropeptides from Gram-negative bacteria using 16S rRNA gene sequencing and mass spectrometry analysis of the HDM extract. These data suggest that airway inflammation might be induced by specific muropeptides in the HDM extract.

In our study, we observed cardinal features of allergic airway inflammation when we assessed a mouse asthma model using ampicillin-treated *D. farinae*. Further, HDM-specific IgE, IgG1, and IgG2 levels were elevated, similar to the ampicillin-untreated HDM asthma models (Figure 3). The expression of ICAM-1 and VCAM-1, which are known to be upregulated in inflamed bronchial endothelial cells, also increased in both groups [41, 42]. In a previous study, the lung function phenotype in mice challenged with *D. pteronyssinus* extracts with high or low LPS was largely similar, but there were differences in the responses at the molecular level in the lung transcriptome study, which were consistent with our findings [43].

We developed a culture method for HDM that kept the bacterial level low for over 18 weeks using ampicillin. Antibiotics were administered to the mites' feed once, and the antibiotic concentration was continuously diluted through multiple passages. As major allergens, Der f 1 and Der f 2 were not reduced in the developed bacteria-reduced HDM extract, and the extract showed allergenicity in the mouse model; therefore, it could be safely used for allergy immunotherapy that required repeated administration of the HDM extract.

In our study, the production of Der f 1 and Der f 2 did not differ between ampicillin-treated *D. farinae* and untreated *D. farinae*. However, in case of the storage mite *T. putrescentiae*, the allergen expression varies across the mite population from different origins, and this variation may be related to differences in the microbiome composition among the members [16]. This implies that due to symbiotic microbes, the mite population can produce different levels of allergens despite belonging to the same species. As mite microbiome composition may be an important factor in allergen production, further studies are needed to investigate the relationship between antibiotic treatment and HDM allergens including trypsin (Der f 3), which is associated with TLR signaling and secretion of IL-6 and IL-8 [44, 45].

The limitation of this study is that the culture period of HDM was 18 weeks; hence, whether the reduction of bacteria and change of allergen production in HDM were maintained thereafter could not be investigated. In the future, it will be necessary to confirm whether the bacterial reduction persists for a prolonged period. In addition, future studies should be conducted to determine whether the HDM extract having low bacterial content has improved efficacy as an immunotherapeutic agents.

In conclusion, we developed *D. farinae* with a low bacterial content, which was sufficient to induce allergic sensitization and immune responses. This study suggests new avenues for the development of allergy immunotherapeutic agent.

## Data Availability

All the data supporting the findings of this study are available within the article and its supplementary information files.

## Additional Points

*Key Points*. (a) Bacterial content in *Dermatophagoides farinae* was reduced by ampicillin treatment. (b) The mouse asthma model was induced properly using ampicillin-treated *D. farinae*. (c) This method would be used to develop more controlled allergy immunotherapeutic agents.

## Supplementary Materials

Supplementary Materials Figure S1: Immunohistochemistry with (a) anti-ICAM-1 and (b) anti-VCAM-1. Data are presented as representative results from at least four independent experiments. D.f.: untreated *Dermatophagoides farinae*; D.f. + AMP: ampicillin-treated *D. farinae*; ICAM-1, anti-intercellular adhesion molecule-1; VCAM-1, vascular cell adhesion protein-1.
