# Supplementary material for: Production of Dermatophagoides farinae Having Low Bacterial Content Using Ampicillin
Source: J Immunol Res. 2023 May 18;2023:9024595. doi: 10.1155/2023/9024595 (PMC10212681; doi:10.1155/2023/9024595)

**Supplementary Materials**

Figure S1. Immunohistochemistry with (a) anti-ICAM-1 and (b) anti-VCAM-1. Data are presented as representative results from at least four independent experiments. *D.f.: untreated *Dermatophagoides farinae*; D.f.+AMP: ampicillin-treated *D. farinae*. ICAM-1, anti-intercellular adhesion molecule-1; VCAM-1, vascular cell adhesion protein-1


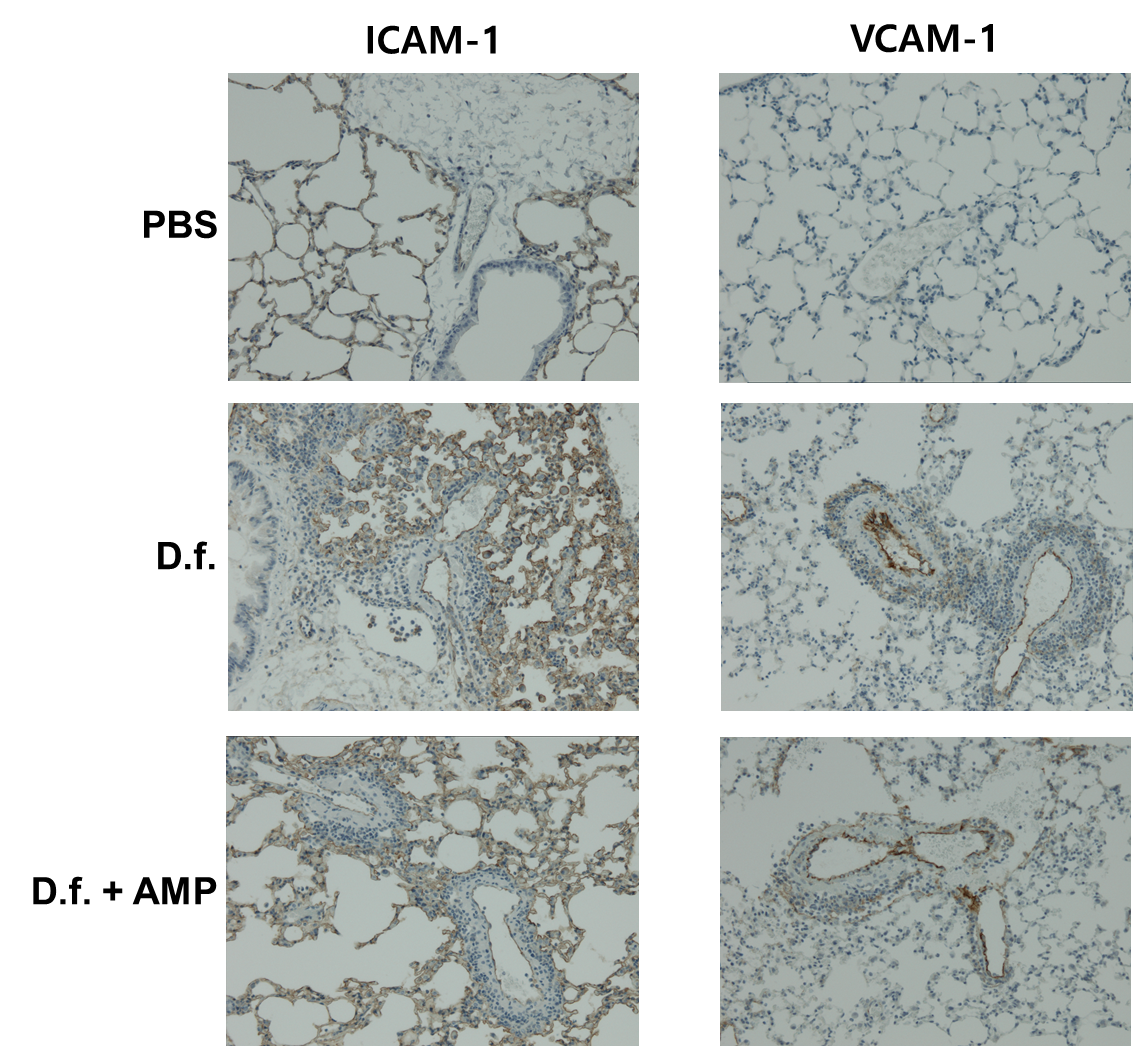

Supplement: Supplementary Materials — Figure S1: Immunohistochemistry with (a) anti-ICAM-1 and (b) anti-VCAM-1. Data are presented as representative results from at least four independent experiments. D.f.: untreated Dermatophagoides farinae; D.f. + AMP: ampicillin-treated D. farinae; ICAM-1, anti-intercellular adhesion molecule-1; VCAM-1, vascular cell adhesion protein-1. [file 9024595.f1.docx]
